# Selectable marker independent transformation of recalcitrant maize inbred B73 and sorghum P898012 mediated by morphogenic regulators *BABY BOOM* and *WUSCHEL2*

**DOI:** 10.1007/s00299-017-2169-1

**Published:** 2017-07-05

**Authors:** Muruganantham Mookkan, Kimberly Nelson-Vasilchik, Joel Hague, Zhanyuan J. Zhang, Albert P. Kausch

**Affiliations:** 1 Plant Transformation Core Facility, Division of Plant Sciences, University of Missouri, 1-33 Agriculture Building, Columbia, MO 65211 USA; 2 Department of Cell and Molecular Biology, University of Rhode Island, 530 Liberty Lane, West Kingston, RI 02892 USA

**Keywords:** Maize, Sorghum, Morphogenic regulators, *BABY BOOM*, *WUSCHEL2*

## Abstract

**Key message:**

**Discriminatory co-expression of maize** ***BBM*** **and** ***WUS*** **transcriptional factor genes promoted somatic embryogenesis and efficient** ***Agrobacterium*** **-mediated transformation of recalcitrant maize inbred B73 and sorghum P898012 genotypes without use of a selectable marker gene.**

**Abstract:**

The use of morphogenic regulators to overcome barriers in plant transformation is a revolutionary breakthrough for basic plant science and crop applications. Current standard plant transformation systems are bottlenecks for genetic, genomic, and crop improvement studies. We investigated the differential use of co-expression of maize transcription factors *BABY BOOM* and *WUSCHEL2* coupled with a desiccation inducible *CRE*/lox excision system to enable regeneration of stable transgenic recalcitrant maize inbred B73 and sorghum P898012 without a chemical selectable marker. The PHP78891 expression cassette contains *CRE* driven by the drought inducible maize *RAB*17_M_ promoter with *lox* P sites which bracket the *CRE, WUS,* and *BBM* genes. A constitutive maize *UBI*_*M*_ promoter directs a ZsGreen *GFP* expression cassette as a reporter outside of the excision sites and provides transient, transgenic, and developmental analysis. This was coupled with evidence for molecular integration and analysis of stable integration and desiccation inducible *CRE*-mediated excision. *Agrobacterium*-mediated transgenic introduction of this vector showed transient expression of *GFP* and induced somatic embryogenesis in maize B73 and sorghum P898012 explants. Subjection to desiccation stress in tissue culture enabled the excision of *CRE, WUS,* and *BBM,* leaving the *UBI*_M_::*GFP* cassette and allowing subsequent plant regeneration and *GFP* expression analysis. Stable *GFP* expression was observed in the early and late somatic embryos, young shoots, vegetative plant organs, and pollen. Transgene integration and expression of *GFP* positive T_0_ plants were also analyzed using PCR and Southern blots. Progeny segregation analysis of primary events confirmed correlation between functional* GFP* expression and presence of the* GFP* transgene in T_1_ plants generated from self pollinations, indicating good transgene inheritance. This study confirms and extends the use of morphogenic regulators to overcome transformation barriers.

## Introduction

Cereal crops arguably feed the world and are grown in greater quantities on more land across more diverse ecosystems than any other crop (Borlaug 2002; FAOSTAT 2012). Over the past three decades, the ability to accomplish heritable genetic transformation of plants has been essential to basic science in plant biology and to myriad crop applications essential to worldwide agriculture and world food supply. Stable genetic transformation of the major cereal crops, such as specific varieties of maize, rice, wheat, and barley (Casas et al. 1993; Cheng et al. 1997; Dai et al. 2001; Gordon-Kamm et al. 1990; Hiei et al. 1997; Ishida et al. 1996; Ritala et al. 1994; Somers et al. 1992; Vasil et al. 1992; Zhao et al. 2000; Popelka and Altpeter 2003; Tingay et al. 1997) by standard technologies has been achieved, but major obstacles remain for genotypes important to genomic analyses and further crop applications. Now, the ability to conduct genome editing in plants (Altpeter et al. 2016; Svitashev et al. 2016) has created an increased need for more efficient and genotype-independent plant transgenic biology. To become more broadly significant, a pan-application systems approach to plant biology and genomics is now being actuated (Altpeter et al. 2016). However, these efforts have been stymied by technological limitations because of inherent features of standard plant transgenics. Plant transformation has been encumbered by several bottlenecks (Altpeter et al. 2016) including genotype and varietal dependence, explant sources and specific cell culture dependence, as well as laborious, expensive, and time-consuming technologies. This is especially true for the cereal crops. In addition, standard transformation biology involves the random stable insertion of a transgene into the genome with the use of a selectable marker transgene. The process to produce and analyze standard stable plant transgenics can involve years of effort, backcrossing into relevant germplasm, intensive analysis, and costly infrastructure. In addition, to apply the science commercially occupies massive resources and funds dedicated to regulatory approval. The need to overcome these obstacles is apparent (Altpeter et al. 2016).

These bottlenecks include numerous practical key factors for transformation of monocots whether *Agrobacterium*, biolistic, or protoplast mediated. These obstacles comprise extensive tissue culture genotype dependence, plant age and type of target tissue, types of vectors, promoters and other UTRs, preferred codon usage, reliance upon and type of selectable marker, *Agrobacterium* strains (when used), inoculation and co-cultivation conditions, and plant regeneration processes. Crucially, obviating these impediments involves achieving genotype and explant independence. Early studies suggested that embryogenic cultures or immature embryo explants are the most suitable and are commonly used for cereal crop transformation (Gordon-Kamm et al. 1990; Hiei et al. 1994; Ishida et al. 1996; Luppotto et al. 1999; Negrotto et al. 2000; Frame et al. 2002; Huang et al. 2004; Huang and Wei, 2005; Hiei et al. 2006; Vega et al. 2008). However, these techniques usually rely upon specific genotypes (such as Hi type II in maize, Bobwhite in wheat, *Nipponbare* in rice, etc.) to achieve successful transformation. Genotypes such as the maize inbred B73 and various sorghum cultivars remain either totally recalcitrant to transformation or transformation that is so inefficient as to be impractical. B73 is of particular interest as it provides the reference maize genome and has a significant history as an important genetic resource for breeding and genomic studies. While one report showed transformation of B73 via meristem culture, the transformation frequency was very low (Zhang et al. 2002) and this procedure has not become routine. Likewise, sorghum transformation by standard techniques is possible but at low frequencies and is also genotype and explant dependent.

Multiple molecular, biological, and developmental reports have shown that several morphogenic genes are involved in plant cell division, somatic embryogenesis, organogenesis, and plant regeneration. Expression of somatic embryogenesis receptor-like kinase1 (*SERK1*) (Schmidt et al. 1997), Leafy cotyledon1 (*LEC1*) and *LEC2* (Harada 2001), Baby boom (*BBM*) (Boutilier et al. 2002), Maize ovule developmental protein 2 (*ODP2*) (Svitashev et al. 2016), Agamous-like15 (Harding et al. 2003), and *WUSCHEL* (*WUS2*) (Zuo et al. 2002; Bouchabke-Coussa et al. 2013) demonstrates morphogenic control of plant development. Recently, a technological breakthrough has been made by selectively and differentially co-expressing morphogenic regulators *BBM* and *WUS2* genes, leading to successful direct somatic embryogenesis from various explant sources of several monocot crops including commercial maize inbred lines, sorghum, rice, and sugarcane (Lowe et al. 2016). Specific overexpression of morphogenic regulators has also recently been shown to improve monocot transformation in several commercial genotypes (Lowe et al. 2016) and facilitate gene editing (Svitashev et al. 2016).

*BBM* encodes an AP2/ERF transcription factor involved with root, seed, basal embryo, and shoot meristem development, and was identified first via subtractive hybridization in *Brassica napus* embryogenic microspore-derived cultures (Boutilier et al. 2002). Boutilier et al. showed that overexpression of *BBM* in transgenic *Arabidopsis thaliana* resulted in the development of ectopic somatic embryos. *BBM* overexpression in *Populus tomentosa* (Deng et al. 2009) and *Theobroma cacao* (Florez et al. 2015) has also been shown to promote somatic embryogenesis. The *Pennisetum squamulatum* apospory specific genomic region of *PsASGR*-*BBML* was shown to promote embryo formation without fertilization in a sexual tetraploid plant (Conner et al. 2015). More recently, in monocots, transgenic expression driven by a constitutively expressed *BBM* transcription factor was observed to confer its influence in a cell-autonomous manner (Lowe et al. 2016). These studies indicate that *BBM* may play a general role in the maintenance of meristematic stem cells in an undifferentiated state, and provide the basis for improved transformation.

*WUS* is a homeodomain-containing transcription factor, which has been shown to be required for stem cell specification in plants and is involved in early embryogenesis, organogenesis, and flowering (Laux et al. 1996). When ectopically expressed, *WUS* promotes somatic embryogenesis and enlarged meristems. The expression of *WUS* is restricted to a subset of meristematic cells subtending the stem cells during all stages of embryogenesis. This expression pattern may indicate that the WUS protein is involved with maintenance in cell fate by a diffusion gradient acting in a non-cell-autonomous fashion. Ectopic expression of *WUS* may facilitate morphogenic alterations in plant development.

Lowe et al. (2016) demonstrated that using a combination of *BBM* and *WUS2* overexpression, they could overcome genotype dependence, and to some extent, explant dependence for transformation of selected genotypes of maize, sorghum, rice, and sugar cane. Their results showed a strong dependence on the promoters used to drive the morphogenic regulators *BBM* and *WUS2.* Using a strongly expressing promoter (Oleosin) in conjunction with *WUS2* led to chimeric and often necrotic callus which regenerated only non-transformed plants. Using a combination strategy of pairing the strongly expressed maize Ubiquitin (*UBI*_*M*_) promoter (Christensen et al. 1992) with *BBM* and a weakly expressed nopaline synthase (*NOS*_*AT*_) promoter from *Agrobacterium tumefaciens* with *WUS2* allowed for stable homogeneous embryogenic callus formation facilitated by phosphinothricin selection. This vector also contained *CRE* driven by the desiccation inducible *RAB*17_M_ promoter with loxP sites bracketing the *CRE, WUS,* and *BBM* transgenes. CRE (Creates Recombination) is a well-studied approach to site-specific recombination involving the loxP sites (Odell et al. 1990). Desiccation-induced expression of *CRE* resulted in the excision of the *CRE*:*WUS*:*BBM* cassette allowing subsequent plant regeneration.

The present study aimed to confirm and extend the previous reports using morphogenic regulators to establish genetic transformation of B73 and sorghum P898012 via co-expression of maize *WUS2* and *BBM* genes without the use of an exogenous selective agent. This study also shows increased frequency of transformation of recalcitrant varieties using this approach.

## Materials and methods

### Plant material

B73 and sorghum public genotype P898012 seeds were obtained from Germplasm Resources Information Network (GRIN: http://www.ars-grin.gov/). Seeds were planted in 3-gallon pots using Pro-mix soil and greenhouse grown at 28/21 °C, and a 16-h light/8-h dark photoperiod. Plants were self-pollinated, and maize ears or sorghum immature embryo explants were collected 9–11 days after pollination. Additional explants including young leaves from aseptically grown seedlings and whole imbibed seeds were also used.

### Morphogenic vector design and *Agrobacterium* strains

**Fig. 1:**
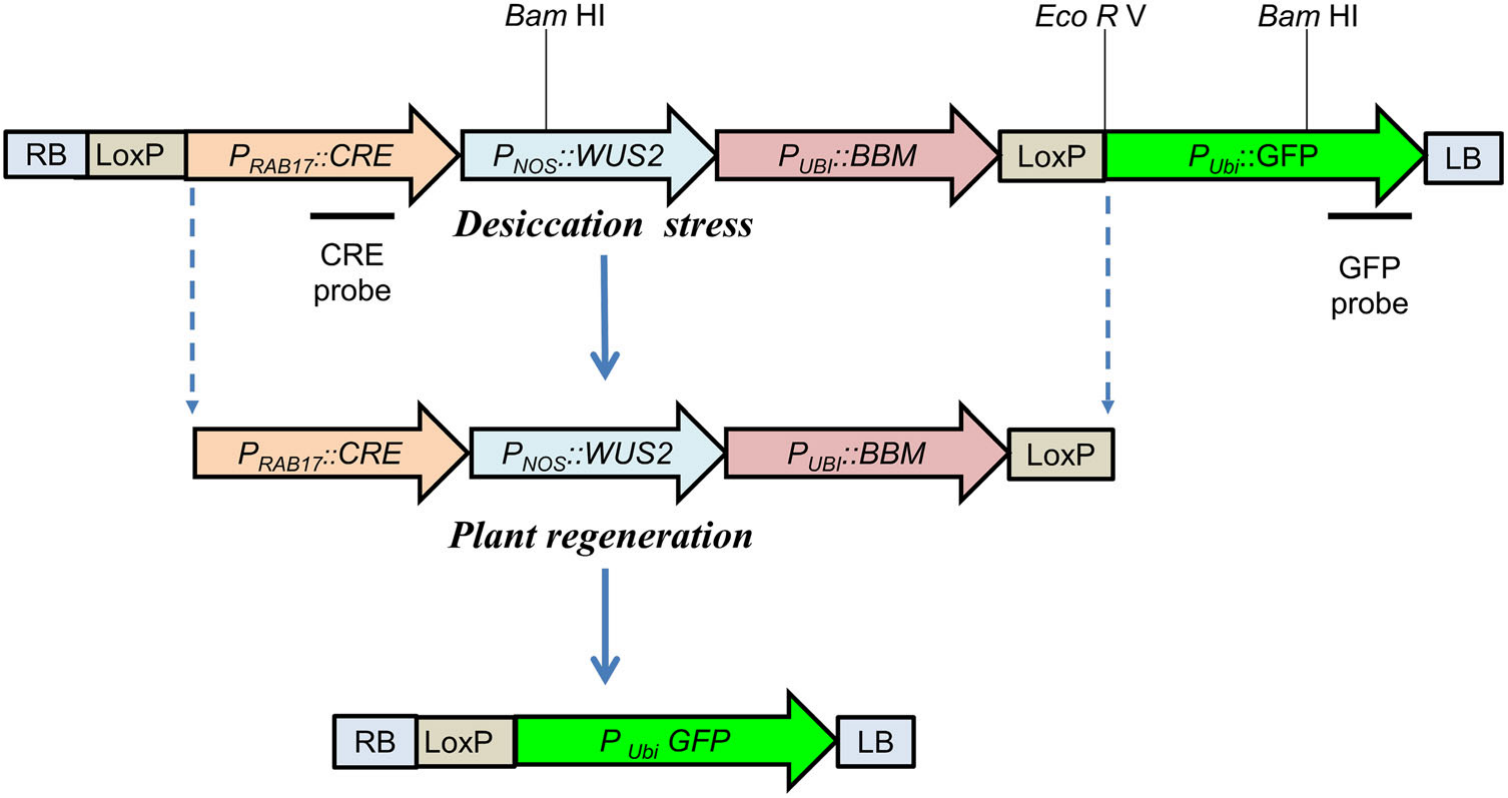
PHP78891 vector. PHP78891 vector comprises four expression cassettes: (1) a *RAB*17_M_: *CRE*; (2) a *NOS*_At_:*WUS2*; (3) *UBI*_M_:*BBM;* and (4) *UBI*_M_: GFP. The *CRE*:*WUS2*:*BBM* cassette is bracketed by *lox* P sites. One *lox* P site is flanked by *Agrobacterium* T-DNA right border (RB) and the other flanks the *UBI*_M_: GFP cassette within *Agrobacterium* T-DNA left border (LB)

Figure 1 shows a diagrammatic representation of the PHP78891 vector. The PHP78891 vector comprises four expression cassettes: (1) RAB17_M_:*CRE*; (2) NOS _At_:*WUS2*; (3) UBI_M_:*BBM;* and (4) UBI_M_:GFP. The *CRE:WUS2:BBM* cassette is bracketed by lox P sites. One lox P site is flanked by the right *Agrobacterium* border and the other flanks the *UBI*_M_:GFP cassette within the left *Agrobacterium* border.

*Agrobacterium tumefaciens* strains AGL1 and EHA 101 were used for maize and sorghum transformation, respectively. The AGL1-SVB strains harbor the PHP78891 binary vector as well as the super-binary vector pTOK233 (Hiei et al. 1994). The AGL1 strain without PHP78891 was used as a negative control for B73 experiments; the AGL1-SVB strain and AGL1 harboring just PHP78891 were used in comparative B73 transformation experiments. EHA 101 harboring the PHP78891 plasmid was used in all experiments on sorghum. PJLU13 was used as a negative control for GFP expression in sorghum and does not contain the *lox* P: *CRE: WUS:BBM: lox* P cassette. PJLU13 has a ubiquitin_Rice_ promoter driving expression of a GFP gene and a nopaline synthase termination signal. Another negative control was PYU2593 consisting of ubiquitin_Maize_ promoter driving a *GFP* gene with a nopaline synthase termination signal.

### Plant transformation

Plant transformation for maize and sorghum followed published protocols (Vega et al. 2008; Do et al. 2016 for maize and sorghum, respectively) with minor modifications.

### *Agrobacterium* culture growth, inoculation, and co-cultivation

For both B73 and P898012 transformation, 5 days before co-cultivation, *Agrobacterium* AGL1 or EHA101 glycerol stocks were streaked onto YEP medium (5 g/L yeast extract, 10 g/L peptone, 5 g/L NaCl, 15 g/L agar, pH 7.0) plates with appropriate antibiotics and incubated at 28 °C in the dark for 3 days. Single colonies were selected and streaked onto fresh YEP plates with the same antibiotics and incubated for 2–3 days. One loop of bacterial culture was suspended in 5 mL of liquid infection medium (Zm-1 for maize, Sb-1 of sorghum) (Tables 1, 2, respectively). Inoculated cultures were shaken at 100 rpm for 4 h at room temperature. Cell density was adjusted to optical density (OD) = 0.35–0.40 at 550 nm using inoculation media. Isolated immature embryos (50–70 per tube) were washed with liquid medium, and replaced with *Agrobacterium* suspension (1 mL) and incubated for 2–5 min. After inoculation, embryos were poured on to the Zm-2 for maize or Sb-2 for sorghum medium plates (Tables 1, 2, respectively). Excess liquid medium was removed thoroughly from the co-cultivation plates, and the embryos were orientated adaxial (scutellar) side up and incubated at 20 °C (for maize) or 25 °C (for sorghum) in darkness for 3 days. Embryos were transferred to Zm-3 (for maize) or Sb-3 (for sorghum) resting medium (Tables 1, 2) and cultured for 7 days. B73 calli were subcultured to fresh Zm-4 medium (Table 1) biweekly whereas P898012 calli were subcultured every 10 days to fresh Sb-4 medium (Table 2). Neither the maize or sorghum media included a selective agent.

**Table 1:** Medium compositions for maize B73 experiments

Medium name and number	Medium name	Media components
Zm-1	Inoculation	N6 salts 4.0 g/L (Chu et al. 1975), sucrose 68.5 g/L, glucose 36.0 g/L, l-proline 700 mg/L, 2,4-D 1.5 mg/L, MES 0.5 mg/L, pH 5.2, Eriksson’s vitamins mix (100×) 1 mL, thiamine HCL 1 mg/L and acetosyringone 1-mL from 100-mM concentration
Zm-2	Co-cultivation	N6 salts 4.0 g/L, sucrose 30 g/L, l-proline 700 mg/L, 2,4-D 1.5 mg/L, MES 0.5 mg/L, pH 5.8, gelrite 3 g/L, Eriksson’s vitamins (100×) 1 ml, Myo-inositol 100 mg/L and acetosyringone 1 mL from 100- mM concentration
Zm-3	Resting	N6 salts 4.0 g/L, sucrose 30 g/L, l-proline 700 mg/L, 2,4-D 1.5 mg/L, MES 0.5 mg/L, pH 5.8, gelrite 3 g/L, Eriksson’s vitamins (100×) 1 ml, SH vitamins (100×) 1 ml, Myo-inositol 100 mg/L, *cefotaxime 250-mg/L * prepared fresh and added after autoclave
Zm-4	Somatic embryo development	MS salts (Murashige and Skoog 1962) 4 g/L, N6 salts macro (10×) 6 mL/L, B5 micro (10×) 6 mL/L, (ref.) sucrose 20 g/L, glucose 10 g/L, l-proline 700 mg/L, 2,4-D 0.5 mg/L, MES 0.5 mg/L, pH 5.8, gelrite 3 g/L, Eriksson’s vitamins (100×) 1 ml, SH vitamins (100×) 1 ml, Myo-inositol 100-mg/L, *cefotaxime 250 mg/L * prepared fresh and added after autoclave
Zm-5	Desiccation	2 Sterile, dry Whatman #70 mm filter paper sealed with parafilm
Zm-6	Regeneration	MS salts 4.3 g/L, sucrose 40 g/L, pH 5.8, agar 8 g/L, MS vitamins (1000×) 5 mL/L, Myo-inositol 100-mg/L, *cefotaxime 250 mg/L * prepared fresh and added after autoclave
Zm-7	Rooting	MS salts 4.3 g/L, sucrose 30 g/L, pH 5.8, agar 8 g/L, IBA 0.5 mg/L, MS vitamins (1000×) 5 mL/L, Myo-inositol 100 mg/L, *cefotaxime 250-mg/L* prepared fresh and added after autoclave

**Table 2:** Medium compositions for sorghum P898012 experiments

Medium name and number	Medium name	Media components
Sb-1	Inoculation	MS salts 2.15 g/L, sucrose 68.5 g/L, glucose 36.0 g/L, 2, 4-D 1.5 mg/L, casamino acids 1 g/L, B5 vitamins (1000×) 1 ml, pH 5.2 and acetosyringone 1 ml from 100 mM concentration
Sb-2	Co-cultivation	MS salts 2.15 g/L, sucrose 20 g/L, glucose 10 g/L, l-proline 700 mg/L, 2,4-D 2.0 mg/L, MES 500 mg/L, ascorbic acid 10 mg/L, B5 vitamins (1000×) 1 ml, pH 5.8, polyvinylpolypyrrolidone (PVPP) 10 g/L, Agar 8 g/L, and acetosyringone 1-ml from 100-mM concentration
Sb-3	Resting	MS salts 4.33 g/L, sucrose 30 g/L, 2,4-D 2.0 mg/L, MES 500 mg/L, ascorbic acid 10 mg/L, asparagine 150 mg/L, coconut water 100 mL/L, B5 vitamins (1000×) 1 mL/L, pH 5.8, phytagel 2.5 g/L, PVPP 10 g/L, *carbenicillin 200 mg/L, *timentin 150-mg/L* prepared fresh and added after autoclave
S4	Callus induction	MS salts 4.33 g/L, sucrose 30 g/L, MES 500 mg/L, 2,4-D 1.5 mg/L, B5 vitamins (1000×) 1 mL/L, pH 5.8, phytagel 2.5 g/L, PVPP 10 g/L, *carbenicillin 200 mg/L, *timentin 150-mg/L* prepared fresh and added after autoclave
Sb-5	Desiccation	2 Sterile, dry Whatman #70 mm filter paper sealed with parafilm
Sb-6	Embryo Proliferation	MS salts 4.33 g/L, sucrose 30 g/L, MES 500 mg/L, 2,4-D 1.5 mg/L, kinetin 0.5 mg/L, B5 vitamins (1000×) 1 mL/L, pH 5.8, phytagel 2.5 g/L, PVPP 10 g/L, *carbenicillin 200 mg/L, *timentin 150 mg/L* prepared fresh and added after autoclave
Sb-7	Regeneration	MS salts 4.33 g/L, sucrose 60 g/L, MES 500 mg/L, l-proline 700 mg/L, zeatin 0.5 mg/L, IAA 1 mg/L, ABA 0.025 mg/L, TDZ 0.1 mg/L, MS vitamin 5 mL/L (1000×), Myo-inositol 100 mg/L, pH 5.8, agar 8 g/L, PVPP 10 g/L, *carbenicillin 200 mg/L, *timentin 150 mg/L* prepared fresh and added after autoclave
Sb-8	Rooting	MS salts 2.15 g/L, sucrose 30 g/L, NAA 0.25 mg/L, IBA 0.25 mg/L, MS vitamin (1000×) 1 mL/L, pH 5.8, phytagel 2.5 g/L, *carbenicillin 200 mg/L, *timentin 150-mg/L* prepared fresh and added after autoclave

### Somatic embryo development, desiccation, and regeneration

Transient expression was evaluated 3-day post-inoculation. Observable colonies of embryogenic cultures developed 5–10 weeks after inoculation. When embryogenic colonies were 0.5–1.0 cm in diameter, they were assayed for GFP expression, and transferred to petri dishes containing 2 sterile dry 3M Whatman filter papers. The petri dishes were sealed with parafilm and incubated at 25 °C in darkness. After 3 days, the desiccated cultures were transferred to regeneration medium (Table 2, Zm-6 for maize) or returned to resting medium (Table 2, Sb-3 for sorghum) for 1 week, followed by subculture to somatic embryo proliferation medium (Table 2, Sb-6 and then regeneration media (Table 2, Sb-7 for sorghum). Well-formed shoots were transferred to rooting media (Table 1, Zm-7 for maize) and (Table 2, Sb-8 for sorghum). Cultures were maintained at 25 ± 2 °C under a 16-h photoperiod for the regenerations stages. Plantlets with healthy roots were transferred to a greenhouse after 2–3 weeks of acclimatization.

### Molecular analysis by PCR and southern blotting

Genomic DNA was isolated from leaf tissue using the method of Chen and Dellaporta (1994) with minor modifications. To confirm the presence of the GFP transgene (ZsGreen) and the absence of the CRE transgene in the T_0_ population, PCR assays were performed using the KAPA 3G Plant PCR kit (KAPA Biosystems) following the manufacturer’s instructions for PCR using purified DNA. The GFP amplicon is 598 bp and the primers were 5′-CCTGACCAAGGAGATGACCA-3′ (forward)/5′-GTCAGCTTGTGCTGGATGAA-3′ (reverse). The PCR assay for the presence/absence of the CRE transgene utilized the primers 5′-CAGTGAAGACCATCCAGCAA-3′ (forward)/5′-CCTAATGTCCTGGCACCTGT-3′ (reverse) resulting in a 227-bp amplified fragment. PCR conditions were 95 °C 3 min followed by 35 cycles of 95 °C 20 s/58 °C 15 s/72 °C 30 s, followed by a final extension at 72 °C 1 min. Products were visualized on a 1.2% agarose gel. In T_1_ lines, the presence/absence of the GFP (ZsGreen) and CRE transgenes was tested using the above primers in conjunction with KAPA 3G Plant PCR kit (KAPA Biosystems) following the manufacturer’s instructions for direct PCR from leaves. In that case, the PCR conditions were 95 °C 10 min followed by 35 cycles of 95 °C 30 s/57 °C 15 s/72 °C 30 s, followed by a final extension at 72 °C 1 min. Products were visualized on a 1.2% agarose gel. For Southern analysis, 15 µg of gDNA was cut overnight at 37 °C using either *Eco*RV or *Bam*HI (New England Biolabs, Ipswitch, MA, USA), both of which cut only once inside the loxP to *Agrobacterium* LB interval found on the transformation plasmid PHP78891 (see Fig. 1). Southern transfer was performed following standard high-salt procedures; GFP and CRE probes were synthesized using the primers discussed above in conjunction with the PCR DIG Probe Synthesis Kit (Roche Applied Science, Indianapolis, IN, USA) according to the manufacturer’s instructions. The pre-hybridization, hybridization, post-hybridization washes, and chemiluminescent detection procedures were all performed according to the manufacturer’s instructions (Roche Applied Science, Indianapolis, IN, USA).

### GFP fluorescence observations

GFP fluorescence observations were made using a Leica M205 FA stereomicroscope supplied with a DFC 7000T camera with GFP filter excitation wavelengths 470–540 nm, emission wavelengths 525–550 nm, and the magnification 10–40× with different filters suitable for the spectrum of 450–490 in excitation of GFP for maize or a Zeiss Discovery v20 with the magnification 10–40× and a GFP 470 filter for sorghum.

## Results

### Transient expression

**Fig. 2:**
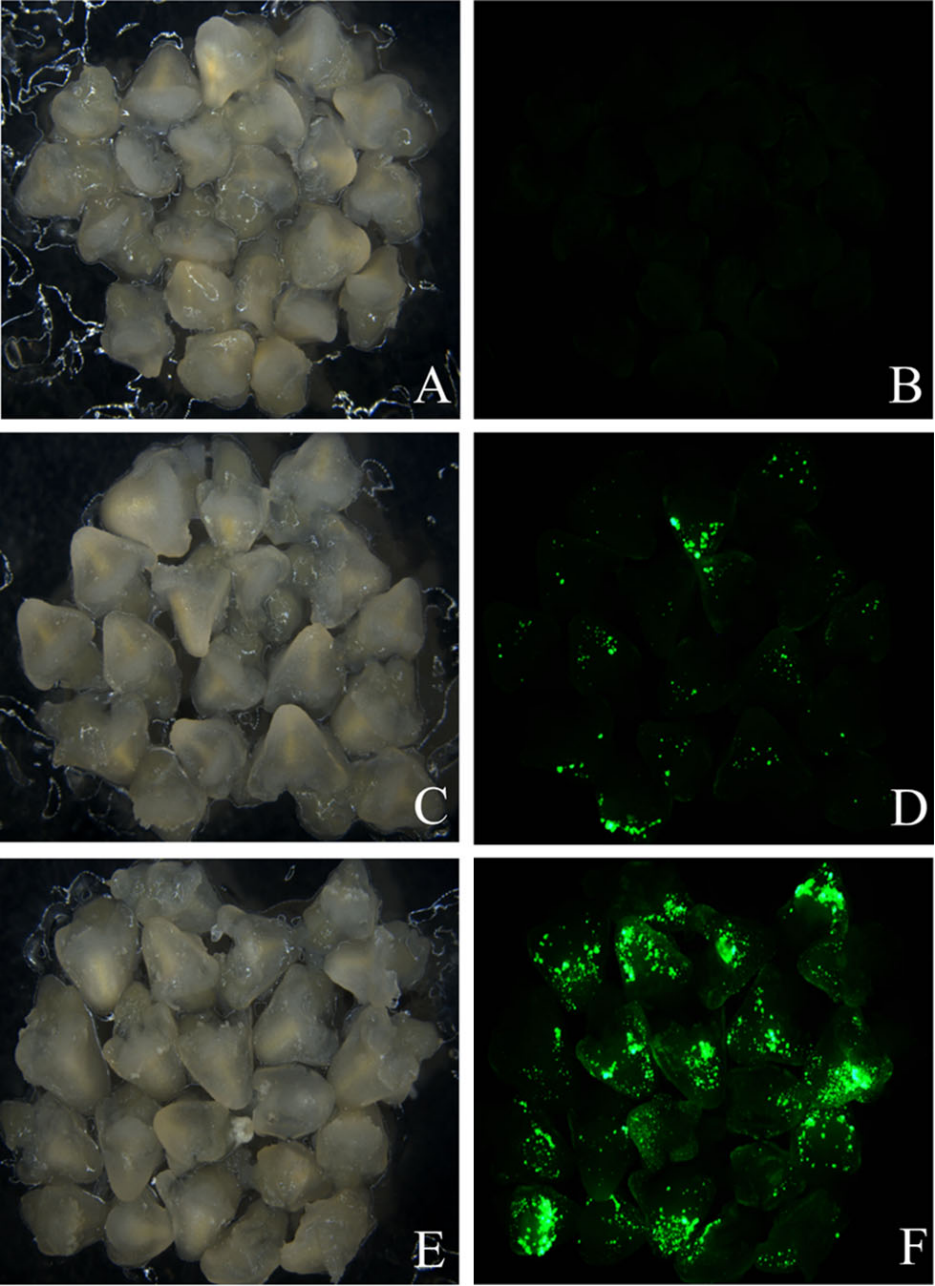
Transient expression of *GFP* in maize B73 using PHP78891. **a** AGL1/empty vector control brightfield image and **b** GFP fluorescence image (negative control). **c** AGL1/PHP78891 brightfield image and **d** GFP fluorescence image showing adaxial surface of the scutellum. **e** AGL1/PHP78891-SBV containing the super-binary vector brightfield image and **f** AGL1/PHP78891-SBV showing GFP foci on the surface of the scutellum cells. Micrographs taken 3 days post-inoculation

Transient expression of *GFP* was observed 3–13-day post-inoculation in B73 and P898012 immature embryos. Transient expression analysis was conducted in B73 using *Agrobacterium* strain AGL1 with or without a super-binary vector. Figure 2a–f and Table 3 show results from three different transformation conditions for B73 immature embryos: (1) *Agrobacterium* AGL1 without a binary vector (Fig. 2a, b; negative control); (2) *Agrobacterium* AGL1 harboring PHP78891 (Fig. 2c, d); and (3) *Agrobacterium* AGL1 harboring PHP78891 containing a super-binary vector SBV (Fig. 2e, f). The frequency of transient transformation was analyzed and recorded by examining the *GFP* expression 3-day post-inoculation (Table 3; Fig. 2a–f). Transient expression efficiencies of B73 using PHP78891 combined with the super-binary vector were 98%, greater than that of the PHP78891 without the super-binary vector (68%), whereas no GFP expression was observed in experiments using AGL1 alone (Table 3; Fig. 2a–f). In addition, experiments using AGL1 with the super-binary vector showed the highest transient expression efficiency, with nearly all embryos showing strong GFP expression on all surfaces of the adaxial side of the scutellum and embryo margins (Fig. 2e, f). These results indicate that *Agrobacterium* AGL1 super-binary vector with PHP78891 was superior for delivery and hence was used in all subsequent experiments for stable transformation of B73.

**Table 3:** Transient GFP expression in maize B73 after 3 days of co-cultivation and embryogenic calli recovery with *Agrobacterium* AGL1, AGL1 with PHP78891, or AGL1 with PHP78891 and super-binary vector (All the GFP transient foci are developed into embryogenic callus or embryoids)

Experiment	*Agrobacterium* and constructs	No. of immature embryos infected	GFP transient expression	% of transient expression	No. of embryogenic calli	% of embryogenic calli
1	AGL1	75	0	0	0	0
2	AGL1	70	0	0	0	0
3	AGL1	80	0	0	0	0
1	AGL1 PHP78891	60	41	68.3	41	68.3
2	AGL1 PHP78891	50	36	72	36	72
3	AGL1 PHP78891	65	43	66.1	43	66.1
1	AGL1 PHP78891-SBV	55	53	96.3	53	96.3
2	AGL1 PHP78891-SBV	60	59	98.3	59	98.3
3	AGL1 PHP78891-SBV	80	78	97.5	78	97.5

Transient expression in sorghum P898012 was conducted using *Agrobacterium* strain EHA101 with or without PHP78891. Results were observed over nine experiments 3-day post-inoculation (Fig. 4a). The transient expression efficiency of P898012 using EHA101 with PHP78891 was an average of 170.3 GFP expressing units per embryo. These results indicate that the *Agrobacterium* strain EHA101 with PHP78891 is sufficient for delivery to support stable transformation experiments in sorghum P898012.

### Stable transformation

GFP transient expression typically does not persist longer that 14–19 days in both B73 and P898012 experiments. Efforts to directly follow transient expression of GFP to stable colonies were unsuccessful. Somatic embryos appeared on the surface of adaxial surfaces on B73 and P898012 immature embryos during 7 days on resting medium (Table 1 Zm-3; Table 2 Sb-3, respectively). After 7 days on the resting media, the cultures were examined for *GFP* expression. Early developing embryos were observed as small and club-shaped, which occurred individually. The vector PHP78891-SBV resulted in higher frequencies of somatic embryo induction (98%) than that of PHP78891 alone (68%) in B73 (Table 3). This suggested that all calli showing GFP positive foci turned out to be embryogenic. After 7 days on resting medium, no somatic embryos were observed in the negative controls containing AGL1 without the binary vector. Somatic embryogenesis frequency appears to be correlated with higher transient *GFP* expression (see Fig. 2).

B73 cultures remained on resting medium for 4–7 weeks with subculturing every 2 weeks. Clusters of embryogenic calli formed between 4 and 7 weeks and were observed on various parts of explanted immature embryos. These calli exhibited small early stage somatic embryos and single-celled protrusions. The frequency of B73 embryogenic callus recovery without a selection agent using *Agrobacterium* AGL1, AGL1 with PHP78891, and AGL1 with PHP78891-SVB is shown in Table 3. AGL1 without the PHP78891 vector showed no somatic embryo response and no formation of embryogenic calli. The AGL1 with PHP78891 showed modest levels of embryo response and formation of embryogenic calli (66.1–72%). However, AGL1 with PHP78891-SVB in B73 showed high rates (over 96%) of embryo response and somatic embryogenic calli formation. Transformation frequency in B73 using AGL1 with PHP78891-SBV (Table 4) also showed a high number of embryogenic calli which survived the desiccation treatment and resulted in the regeneration of green shoots with an overall transformation frequency of 14.5–15%. Desiccation stress was applied 5–6-week post-inoculation. After desiccation treatment, callus that was not GFP positive appeared necrotic and did not typically survive subculture (Fig. 2a–d).

**Table 4:** Transformation frequency in maize B73 using AGL1 with PHP78891-SBV

Experiment	No. of immature embryos infected	Embryogenic calli	No. of calli surviving desiccation	No. of GFP positive calli	No. of GFP positive events that regenerated	Transformation frequency (%)
1	55	53	32	29	8	14.5
2	60	59	29	25	9	15
3	80	78	70	63	12	15

Similar results were observed in P898012 experiments. Transient expression after 3 days showed punctate single-celled GFP expression (Fig. 4a). After 13 days, these cultures, showed the presence of immature somatic embryos (Fig. 4b) in stages of development comparable to 4–7-day post-pollination zygotic embryos. These somatic embryos were not entirely GFP positive, indicative of transient expression. Under the conditions of this study, these somatic embryos did not mature, and GFP expression was eventually lost. After 4–7 weeks on resting media, GFP expression developed as small clusters on resting media (Fig. 4c, d). Representative frequency of embryogenic callus recovery with *Agrobacterium* EHA101 in P898012 (Table 5) shows that in comparison to the GFP expressing control without the *CRE*:*WUS*:*BBM* cassette (PJLU13 and PYU 2593 plasmids), embryos inoculated with PHP78891 produced GFP positive embryogenic calli at significantly higher frequencies, up to 54.54%.

**Table 5:** Frequency of GFP expression of PHP78891 without selection using *Agrobacterium* EHA101 in sorghum P898012

Experiment	*Agrobacterium* EHA101 with or without PHP78891	No. of immature embryos infected	GFP positive embryogenic calli	% GFP expression
1	EHA101 PJLU13	11	0	0
2	EHA 101 PYU 2593	55	0	0
3	EHA 101 PYU 2593	46	0	0
4	EHA101 PHP78891	11	6	54.54
5	EHA101 PHP78891	180	28	15.56
6	EHA101 PHP78891	128	30	23.44
7	EHA101 PHP78891	52	10	19.23

**Fig. 3:**
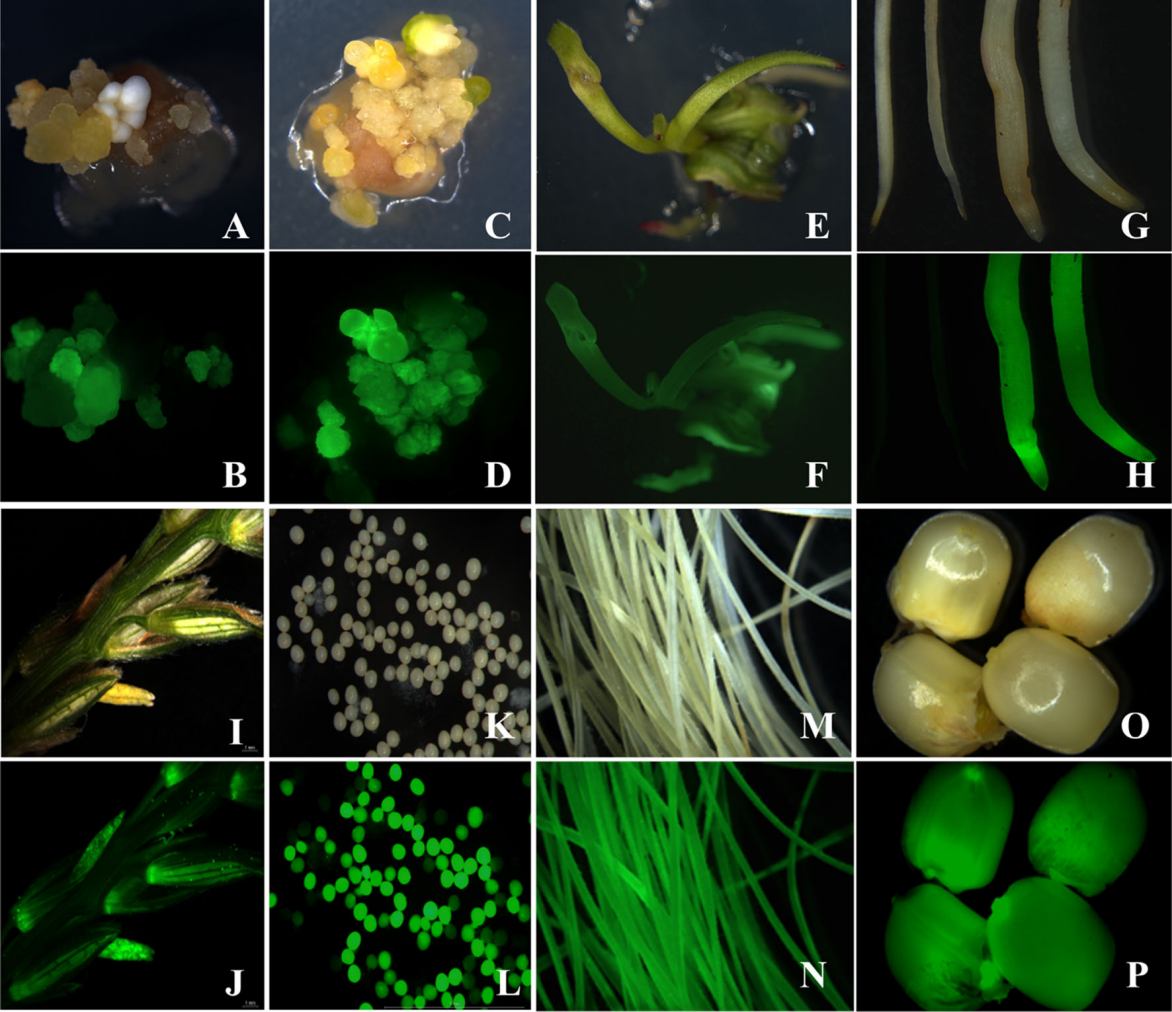
Expression of PHP78891 in T_0_ transgenics of maize B73. Maize B73 AGL1 PHP78891 calli after 3-day desiccation stress calli; comparable **a** (Brightfield image) and **b** (GFP image), AGL1 PHP78891-SBV after 3-day desiccation shows higher GFP sector expression frequencies; **c** (brightfield image) and **d** (GFP image), homogeneously GFP expressing shoot in regeneration after 3 days desiccation; **e** (Brightfield image) and **f** (GFP image), GFP positive roots (*right*) and wild type (*left*); **g** (Brightfield image) and **h** (GFP image), tassel from PHP7889-SBV event after anthesis with GFP positive anthers; **i** (Brightfield image) and **j** (GFP image), isolated pollen showing 1:1 segregation for GFP; **k** (Brightfield image) and **l** (GFP image), silk from regenerated plant; **m** (Brightfield image) and **n** (GFP image), kernels from regenerated plant; **o** (Brightfield image) and **p** (GFP image), kernels from regenerated plant

**Fig. 4:**
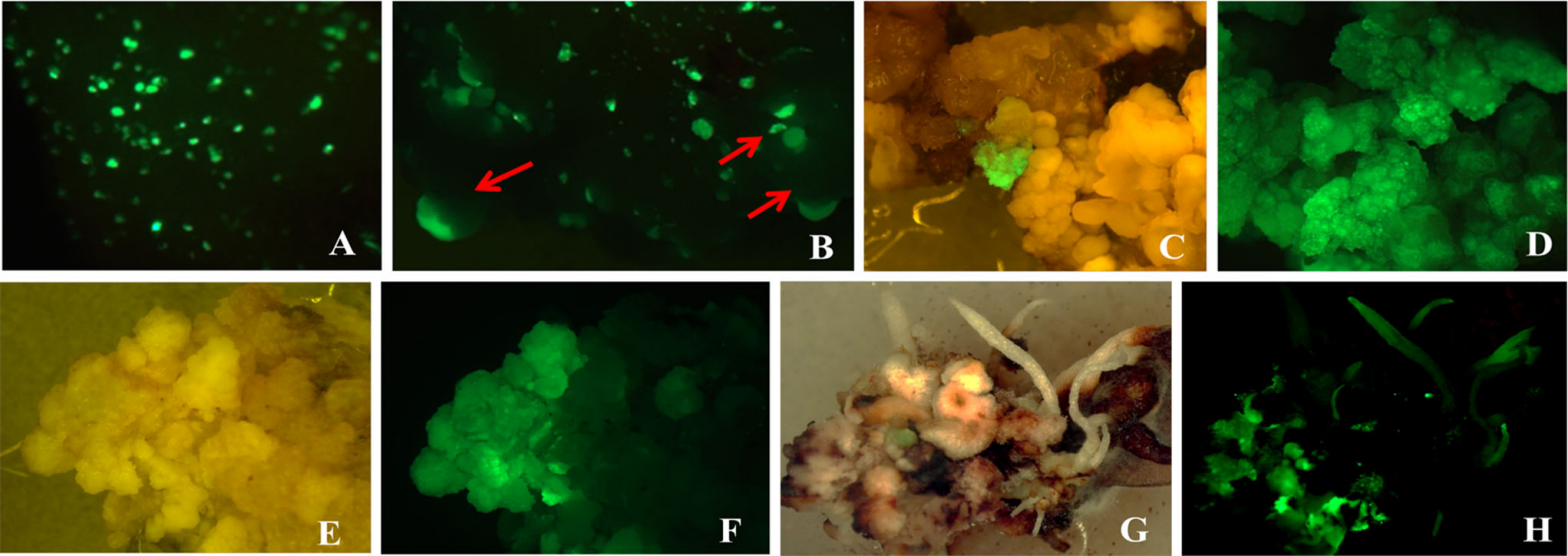
Transient and stable sorghum P898012 Transformation using PHP78891. **a** Transient expression EHA 101 PHP78891 3-day post-inoculation (GFP image); **b** Early stage somatic embryos (*arrows*) 13-day post-inoculation (GFP image). EHA 101 PHP78891 derived events after desiccation shows heterogeneous callus. **c** Brightfield and GFP image shows GFP positive embryogenic cluster within GFP negative organized callus. **d** Homogeneous GFP expressing embryogenic callus before desiccation (brightfield image); **f** corresponding GFP image showing heterogeneous callus after desiccation; **g** Heterogenous GFP expression in callus during plant regeneration (brightfield image); **h** corresponding GFP image

These GFP positive calli were embryogenic (type II) and occurred within organized (type I) calli (Fig. 4). With repeated subculture every 2 weeks, these GFP positive clusters developed into homogenous proliferating somatic embryogenic calli for both maize (not shown) and sorghum (Fig. 4 for sorghum). After embryogenic calli were observed to be GFP positive and growing (5–6-week post-inoculation), selected colonies were placed onto sterile, dry filter paper to induce desiccation. After 3-day desiccation, the calli were dry, shrunken, and had a yellowish appearance. For B73, desiccated calli were subcultured to medium Zm-6 (Table 1). Similar to post-desiccation treatment of maize calli, sorghum did not show uniform *GFP* expression after desiccation stress (Fig. 4e, f). For P898012, desiccated cultures were returned to resting medium (Table 2, Sb-3) for 1 week and then subcultured to somatic embryo development medium (Table 2, Sb-4) for 2 weeks. The resulting proliferating calli showed a heterogeneous *GFP* expression after desiccation (Fig. 3a–d for maize and Fig. 4e, f for sorghum). In B73, the number and frequency of GFP positive calli was higher in those embryos inoculated with the AGL1 PHP78891-SVB (Fig. 3c, d) in comparison to embryos inoculated with AGL1 PHP78891 (Fig. 3a, b). These results are consistent with the transient expression results. All calli were then subcultured to regeneration media (Table 1, Zm-6 for maize; Table 2, Sb-6 for sorghum). Two weeks after subculture to regeneration media, many of the embryos had turned photosynthetically green and produced shoot buds, although some calli sectors or regenerating tissues had turned necrotic and did not survive. The regenerating shoot buds were then transferred to medium Zm-6 (Table 1) for maize or Sb-6 (Table 2) for sorghum for 2–3 additional weeks. After transfer to regeneration media, the calli continued to show a heterogeneous expression of *GFP* (Fig. 3e, f for maize; Fig. 4g, h for sorghum), but the individual plantlets regenerating from the calli appeared homogenous for *GFP* expression for both B73 and P898012.

B73 plants regenerated from these events exhibited *GFP* expression in the T_0_ plants in vegetative tissues and floral structures, including young leaves (not shown), roots (Fig. 3g, h), tassel inflorescences and anthers (Fig. 3i, j), pollen (Fig. 3k, l), silks (Fig. 3m, n), and mature seeds (Fig. 3o, p). Pollen was segregating for 1:1 *GFP* expression. Other tissues including mature leaf surfaces, leaf vascular cell tissue, leaf margins with trichomes, tillers, and prop roots exhibited strong *GFP* expression as well. T_0_ P898012 plants also showed expression of *GFP* in vegetative and floral structures to maturity (data not shown).

**Fig. 5:**
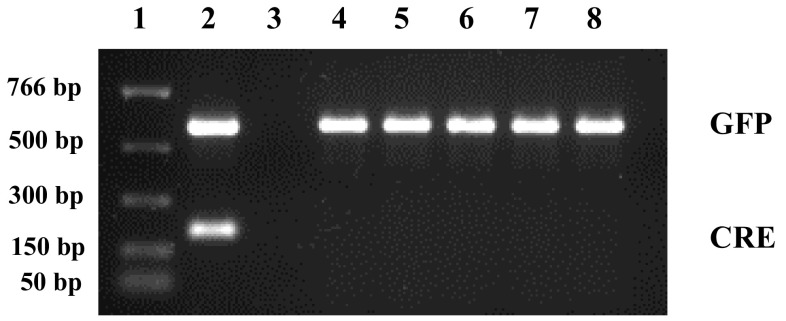
PCR amplification of ZsGreen and* CRE* fragments from maize B73 events. Lanes 1, 300 ng NE Biolabs PCR Marker; lane 2, positive control, amplification of ZsGreen (*upper band*, expected size 594 bp) and Mo CRE (*lower band*, expected size 227 bp; lane 3, B73 wild-type negative control; lanes 4–8, transformed plants, M-1-1, M-1-2, M-1-5, M-1-6, and M-1-8

**Fig. 6:**
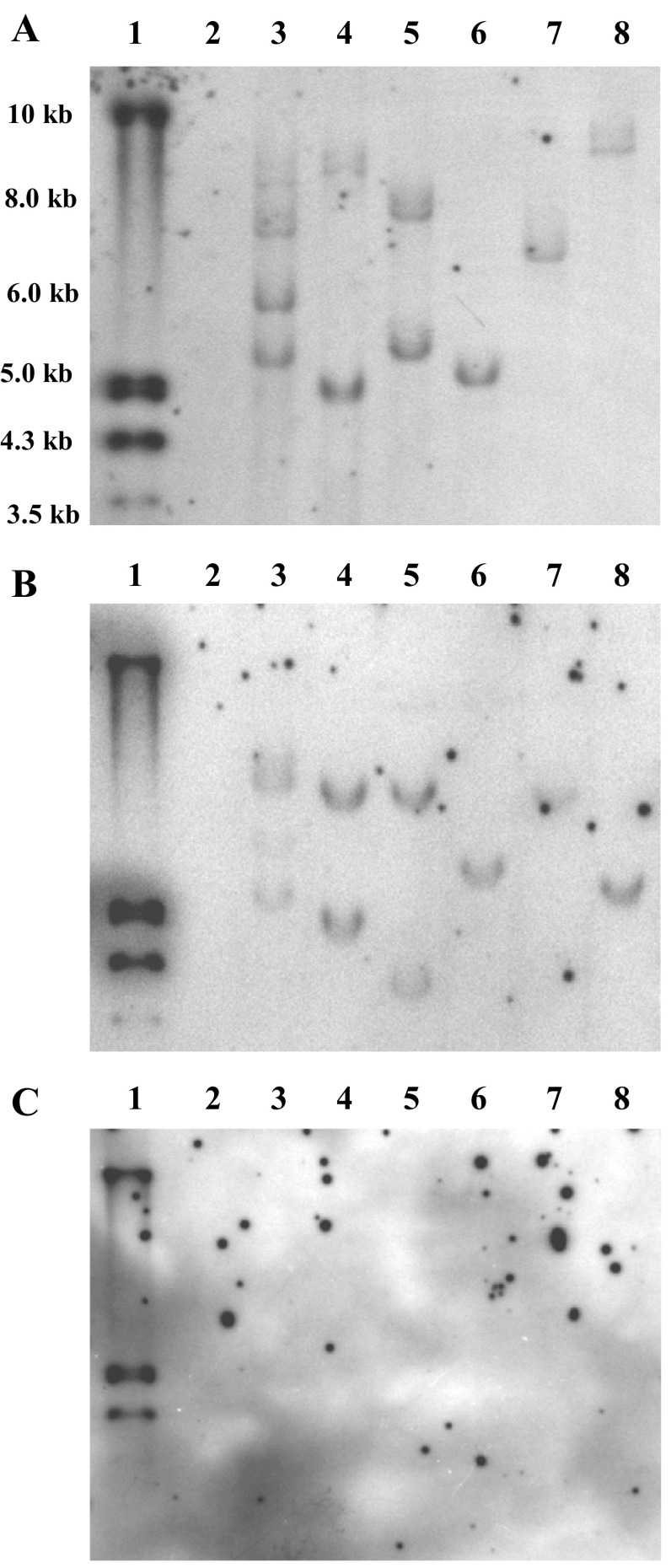
Southern blot of PHP78891 transformed maize B73 events using a DIG-labeled probe.** a** *Eco*RV restriction digest probed for* GFP* as shown in Fig. 1.** b** *Bam*HI restriction digest probed for* GFP*, as shown in Fig. 1.** c** Stripped* Bam*HI restriction digest shown in b, re-probed for* CRE*. Lanes 1, DIG-labeled molecular weight ladder III (Roche Diagnostics Corporation, IN, USA); lane 2, nontransformed maize B73; lanes 3–8, PHP78891-transformed B73 events, i.e., M-1-6, M-1-8, MM-2-9, MM-4-1b, MM-4-1F, and MM-5-0, respectively

Results from molecular analysis confirmed that regenerated B73 and P898012 plants are transgenic. Figure 5 shows PCR amplification of *GFP* and *CRE* transgenes fragments. Transformed B73 plants show the presence of the *GFP* cassette but no amplification of the *CRE* amplicon. This result, in conjunction with successful regeneration of GFP positive plants, indicates the likely excision of the loxP-bound *CRE*:*WUS*:*BBM* cassette in the transformed plants. Southern blots confirmed these results (Fig. 6a–c). *Eco*RV cut once within 5′ region of the *GFP* cassette. Figure 6a shows PHP78891-transformed B73 events; M-1-6, M-1-8, MM-2-9, MM-4-1b, MM-4-1F, and MM-5-0; l-r, in lanes 3–8 (respectively). At least three of these events (MM-4-1b, MM-4-1F, and MM-5-0; l-r) show a single band when probed for *GFP*. *Bam*HI cut twice within the cassette and when probed for *GFP* also showed the same expected insertions (Fig. 6b). When this blot was stripped and re-probed for *CRE*, these blots showed the absence of the *CRE*:*WUS*:*BBM* cassette (Fig. 6c). These results collectively confirmed the presence of the transgene and excision of loxP flanked cassettes, and that most of the transformants are low copy insertion events (Table 6).

**Table 6:** Frequency of stable embryogenic callus recovery without selection using *Agrobacterium* EHA101 in sorghum P898012

Experiment	*Agrobacterium* EHA101 with or without PHP78891	No. of immature embryos infected	No. of desiccated embryogenic calli	No. of GFP positive events that regenerated	Transformation frequency (%)
1	PJLU13	11	0	0	0.0
2	PYU 2593	55	0	0	0.0
3	PYU 2593	46	0	0	0.0
4	PHP78891	11	6	1	9.1
5	PHP78891	180	28	5	2.8
6	PHP78891	128	30	11	8.6
7	PHP78891	52	10	6	11.5

**Fig. 7:**
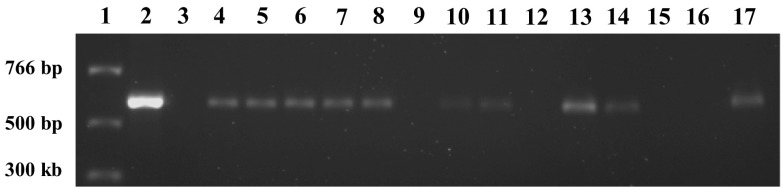
PCR of inheritance and segregation of the GFP transgene in T_1_ plants generated from self pollinations. Shown are PCR analysis of 14 T_1_ plants from the MM-4-1F line. Lane 1, molecular weight markers; lane 2, control reaction for GFP marker; lane 3, wild-type nontransformed control; lanes 4–17, T_1_ plants testing positive for the transgene in lanes 4, 5, 6, 7, 8, 10, 11, 13, 14, and 17; and, negative in lanes 9, 12, 15, and 16

**Fig. 8:**
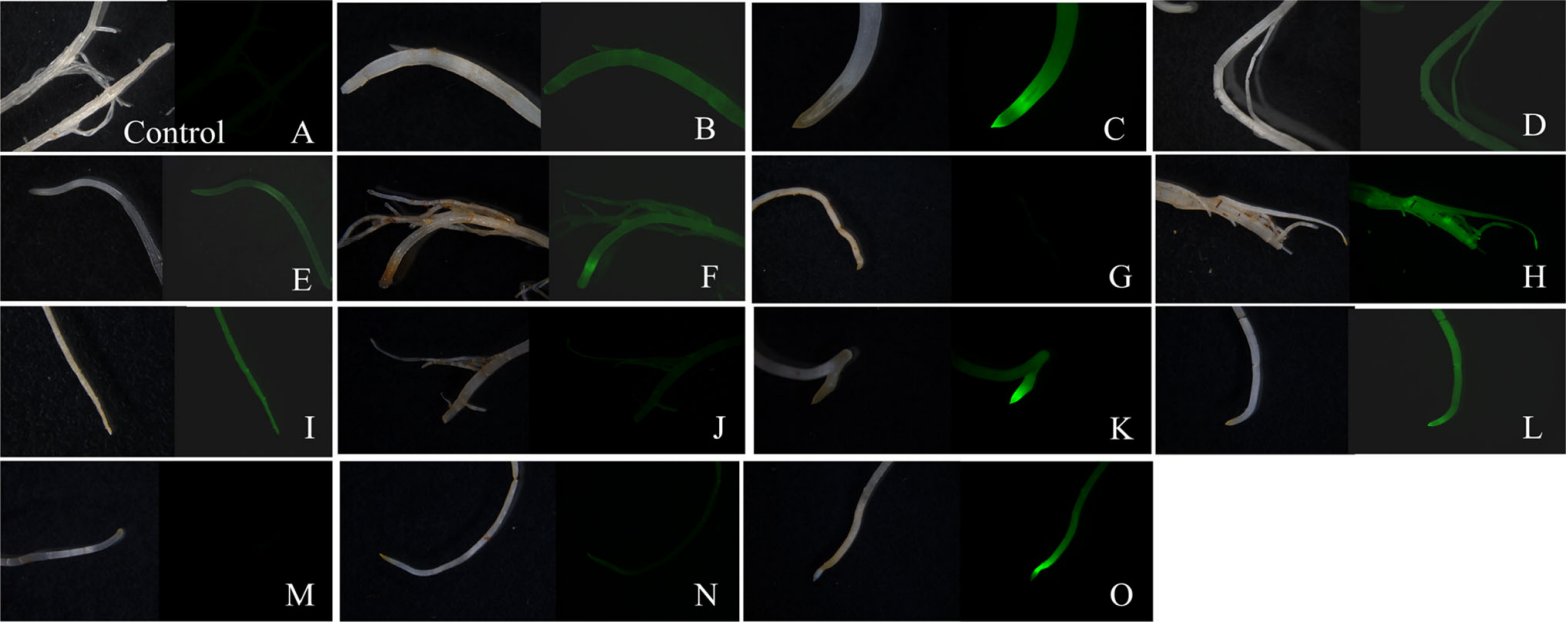
Inheritance and segregation of the functional GFP transgene in T_1_ plants generated from self pollinations. Wild-type control **a** is negative for GFP expression. Roots from T_1_ plants from the MM-4-1F line **b**–**o** are shown in paired brightfield and GFP micrographs corresponding to the PCR results. The T_1_ plants in **b**–**o** correlate directly with lanes 3–17 in Fig. 7

Inheritance and segregation of the transgene were investigated in T_1_ plants generated from self pollinations. PCR results confirmed that the hemizygous transgene was stably inherited in T_1_ plants as a single Mendelian trait (Fig. 7) which correlate with functional GFP expression (Fig. 8; Table 7). Representative PCR analysis of 14 T_1_ plants from the MM-4-1F line showed that nine tested positive for the transgene and four plants tested negative (Fig. 7). Functional GFP analysis confirmed these results with the same plants that were PCR positive for GFP expression and those that tested negative for PCR were also negative for GFP expression. The T_1_ plants in B–O (Fig. 8) correlated directly with the PCR lanes 3–17 in Fig. 7.

**Table 7:** Correlation between functional GFP expression and presence of the GFP transgene in T_1_ plants generated from self pollinations

T_0_ parent line	T_1_ plant ID	GFP expression	PCR analysis
Control	A	Negative	Negative
	B	Positive	Positive
	C	Positive	Positive
	D	Positive	Positive
	E	Positive	Positive
	F	Positive	Positive
MM-4-1F	G	Negative	Negative
	H	Positive	Positive
	I	Positive	Positive
	J	Negative	Negative
	K	Positive	Positive
	L	Positive	Positive
	M	Negative	Negative
	N	Negative	Negative
	O	Positive	Positive

## Discussion

Standard plant transformation is usually *Agrobacterium,* biolistics, or protoplast mediated, and is hampered by several technical bottlenecks (Altpeter et al. 2016) as well as long turnaround times to recover transgenic plants (9–18 months). The current developments in genomics and gene editing have amplified the need for transgenics (Altpeter et al. 2016) and have vastly increased the necessity for solutions to these obstacles. Recent reports (Lowe et al. 2016; Svitashev et al. 2016) using morphogenic regulators to mediate transformation offer a promising breakthrough solution to many of the obstacles which have encumbered standard plant transformation. Lowe et al. 2016 (personal communication, and Lowe SIVB Meetings 2016) reported use of this strategy on many commercial inbreds and using various explant sources including immature embryos, mature seeds, and mature leaves.

Most protocols for standard plant transformation rely entirely upon highly articulated tissue culture regimes for stable gene introduction and plant regeneration, which are genotype specific. These schemes all use specific genotypes which have been worked on for decades and focused on transformation, selection, and transformation responses. The use of morphogenic regulators to overcome barriers associated with these constraints has created a watershed advance for plant genome modification and agriculture. While the use of morphogenic regulators had been previously explored (Gordon-Kamm et al. 2002), differential overexpression of *BABY BOOM* and *WUSCHEL2* driven by independent promoters was demonstrated to surmount many genotype obstacles associated with cell division and somatic embryogenesis during transformation (Lowe et al. 2016). Lowe et al. (2016) show dramatic increases in transformation frequencies through optimal expression of *BBM* and *WUS* and expanded breadth of transformation amenable genotypes. However, even though those studies broadened the range of transformable monocot lines, a genotype-independent transformation approach has remained yet to be achieved. For example, routine transformation of maize B73, despite its importance as a genetic model, has remained elusive.

Embryo formation in plants occurs via multiple mechanisms, such as zygotic embryogenesis, androgenesis, somatic embryogenesis, and apomixes (Mordhorst et al. 1997; Koltunow and Grossniklaus 2003). Several transcription factors are known to play a significant role in cell dedifferentiation and division and may control or induce somatic embryogenesis in plants. As a transcription factor, *BBM* has been identified as a marker for embryogenic cultures (Boutilier et al. 2002; Deng et al. 2009; Florez et al. 2015) and is likely involved with regulation of multiple genes involved with the early embryo development. *WUS* is known to be involved in the early embryogenesis (Laux et al. 1996) and as a homeodomain-containing transcription factor also probably regulates a battery of genes. The use of differentially expressed *BBM* and *WUS* has resulted in effecting ectopic somatic embryogenesis. Lowe et al. (2016) indicate that the transiently expressed WUS protein may be diffusible and capable to influence surrounding cells to be stimulated to divide and that this is consistent with the previous investigations on the WUS protein as a contributor to apical meristem organization (Mayer et al. 1998; Gallois et al. 2002; Yadav et al. 2011, 2013).

Our investigation indicates that transient expression of a *CRE*:*WUS*:*BBM*:*GFP* construct elicits transient *GFP* expression coincident with the early stages of somatic embryogenesis in B73 and P898012. However, following a period of transient expression, stable GFP positive transformed colonies did not appear until 4–7-week post-*Agrobacterium* inoculation. This indicates that the growth of cells from stably integrated events may not originate from transiently expressing cells or that maintained expression of morphogenic regulators requires significant time to elicit their effect. Once established, however, stable transformed the early embryogenic and GFP positive colonies appear within more organized compact (type I) callus. The embryogenicity and *GFP* expression in these events appears homogenous, indicating that they are probably not mosaic for insertion or expression. It is important to note that a selectable marker gene, such as *PAT* (phosphinothricin acetyltransferase), *BAR* (bialphos resistance), *ALS* (acetolactate synthase), *HPT* (hygromycin phosphotransferase), etc., was not used in this procedure. There are few substantiated reports of visual selection of transgenic plants without the use of a selection agent. Recently, Svitashev et al. (2016) showed the use of the maize transcription factors (maize ovule developmental protein 2 (*ODP2*) and maize *WUS*) in conjunction with a selectable and a visible fusion reporter gene marker. The selectable marker was a herbicide resistance transgene, maize-optimized phosphinothricin-*N*-acetyltransferase (*MOPAT*) translationally fused with the visual marker gene red fluorescent protein (*DSRED*). Transformants were generated using biolistic-mediated transformation followed by herbicide selection and plant regeneration. The GFP positive embryogenic calli in the present study could be maintained through continued subculture but would not regenerate. This observation suggests that expression of *BBM* and *WUS* is sufficient and necessary to induce somatic embryogenesis in B73 and P898012. Acting in this way, differential overexpression of morphogenic regulators acts as a type of developmental selectable marker, in that only those cells which are transformed and expressing the gene become embryogenic and arrested in that stage of development. Lowe et al. (2016) also reported the recovery of one inbred maize line without the use of a selectable marker. Thus, expression of *BBM* and *WUS* promotes recovery of stable transformants but prevents further development and plant regeneration. This observation was also intimated by Lowe et al. (2016) and necessitates the need for inducible expression of *BBM* and *WUS* or inducible excision via site-specific recombination.

The *CRE*/*lox* site-specific recombination system of bacteriophage P1 has been used for various gene excision studies in transgenic plants (Dale and Ow 1990; Hoa et al. 2002; Srivastava et al. 1999). Desiccation induction of *RAB*17_M_: *CRE* expression can be used to remove the *WUS2* and *BBM* transcriptional genes from the transgenic embryogenic callus before transfer to the regeneration medium. Desiccation stress was applied after visible GFP calli were readily observable (5–6-week post-inoculation). This indicates that the GFP calli were most likely the result of stable integration events. Following the desiccation treatments for both maize and sorghum, the calli were not uniform for GFP expression, indicating that either desiccation stress or induction of the RAB17_M_ promoter was not uniform, loxP flanked cassette excision was not consistent or a combination of these factors. The *CRE*:*BBM*:*WUS* cassette is flanked by two directly repeated loxP sites. Induced *CRE* expression mediates excision of the cassette allowing plant regeneration. Before transfer to the regeneration medium, the embryos were desiccated for 3 days to eliminate the ectopic expression of *WUS2* and *BBM*, to support normal plant regeneration. Without desiccation, we observed ectopic expression manifesting as secondary clusters of somatic embryos, folded leaves, and very short plantlets with thick abnormal root structures (data not shown).

The desiccation treatment of early embryogenic GFP positive calli likely induces the *RAB*17_M_ promoter and expression of *CRE* resulting in the excision of the *lox P: CRE*:*WUS*:*BBM*: *lox P* cassette. The resulting calli were no longer homogeneous but exhibited large sectors of GFP embryogenic callus which were subsequently capable of regeneration. It is well known that *CRE*-mediated excision can be variable under some situations. Molecular analysis by both PCR and Southern blots confirmed the likely removal of the *loxP:CRE*:*WUS*:*BBM*: *loxP* cassette in regenerated plants. Furthermore, the Southern analysis show presumed single copy insertion events, indicating that these are probably not chimeric mosaics for loxP excision. This observation indicates that the regenerated plants from the current study are derived either from single-celled excision events or multi-celled, but homogenous excision events.

While *Agrobacterium*-mediated transformation is a common method for gene transfer to plants, transformation frequency differs widely among various *Agrobacterium* strains (Wu et al. 2003; Vega et al. 2008; Cho et al. 2014; Do et al. 2016; Zhang et al. 1999), and genotypes. For example in maize using EHA101, the efficiency reported for Hi II by Frame et al. (2002) was 5.5%, 2–6% in H99 (Sidorov et al. 2006), and 12–18% in Hi IIA X B F1 (Vega et al. 2008). Ishida et al. (1996) developed an *Agrobacterium* strain containing a super-binary vector with additional copies of *virB*, *virC*, and *virG* genes for transformation of immature embryos in maize A188. Their experiments showed a high frequency of transformation (5–50%), with all resulting plants being fertile. We have shown a higher frequency of both transient and stable transformation of B73 using the AGL1 strain containing the super-binary vector. Higher transformation frequency also correlated with higher plants recovered after excision of the *CRE*:*WUS*:*BBM* cassette. Wu et al. (2014) also reported a higher frequency of transformation in sorghum using the AGL1 strain containing the super-binary vector.

B73 represents a recalcitrant transformation genotype of genetic and genomic significance. Standard transformation techniques result in 0% transformation. We have shown that use of AGL1 strain containing the super-binary vector increases vector delivery. In conjunction with the overexpression of the morphogenic genes *BBM* and *WUS*, we were able to achieve significant frequencies of stable transformation of B73. Following desiccation, induced excision of the morphogenic genes and high rates of plant regeneration were achieved. This approach enables a significantly increased frequency of transformation from 0 to 15% for B73 and up to 6.2% for P898012 genotypes without the use of selection agents. Selectable marker independent transformation may contribute to overcoming transformation barriers and facilitate studies using gene editing functions. Our study confirms and extends the previous reports using morphogenic regulators to improve transformation (Lowe et al. 2016; Svitashev et al. 2016). In this study, we have established reliable transformation of the recalcitrant B73 and P898012 via co-expression of *BBM* and *WUS*. Using this approach, we also show increased frequency of transformation of these recalcitrant varieties. The observation of early stage somatic embryos 13 days after DNA delivery indicates that this response may be the result of transient expression of *BBM* and *WUS* prior to integration. We expect that these results will be further extended to include explant independence and exploitation of transient expression of the morphogenic regulators.

The ability to deploy gene editing functions, such a zinc finger nuclease (*ZNF*), transcription activator-like effector nucleases (*TALENS*), and more recently, clustered regularly interspaced short palindromic repeats (*CRISPR*), has dramatically increased the need for more efficient and genotype-independent plant transgenic biology. Svitashev et al. (2016) used biolistic delivery for introduction of ribonucleoprotein (RNP) complexes and the use of the maize transcription factors *ODP2* and *WUS*, chemical and visual selection for the validation, and recovery of C*RISPR*/Cas9 target sequences. These studies indicate that the biology and technology for plant transformation is entering a new era. The ability to conduct gene editing, genetic modification, and plant transformation that is genotype-independent, *Agrobacterium* independent, DNA free (non-GMO), tissue culture free, high-throughput, high efficiency, and relatively rapid is of significant importance to basic plant biology and world agriculture. We expect that the current research in this area will achieve these goals.

### **Author contribution statement**

ZZ and AK conceived and designed research; MM, KNV, and JH conducted research; MM, KNV, JH, ZZ, and AK analyzed results; MM, KNV, JH, ZZ, and AK wrote and revised the manuscript.
